# P-cadherin signals through the laminin receptor α6β4 integrin to induce stem cell and invasive properties in basal-like breast cancer cells

**DOI:** 10.18632/oncotarget.1459

**Published:** 2014-01-06

**Authors:** André Filipe Vieira, Ana Sofia Ribeiro, Maria Rita Dionísio, Bárbara Sousa, Ana Rita Nobre, André Albergaria, Angélica Santiago-Gómez, Nuno Mendes, Renê Gerhard, Fernando Schmitt, Robert B. Clarke, Joana Paredes

**Affiliations:** ^1^ IPATIMUP – Institute of Molecular Pathology and Immunology of the University of Porto, Porto, Portugal; ^2^ ICBAS – Institute of Biomedical Sciences Abel Salazar, Porto, Portugal; ^3^ Gulbenkian Program for Advanced Medical Education, Lisbon, Portugal; ^4^ Breast Biology group, Institute of Cancer Sciences, University of Manchester, Manchester UK; ^5^ Faculty of Medicine of the University of Porto, Porto, Portugal

**Keywords:** P-cadherin, Breast cancer, α6β4 integrin, cancer stem cells, invasion, FAK, Src

## Abstract

P-cadherin is a classical cell-cell adhesion molecule that, in contrast to E-cadherin, has a positive role in breast cancer progression, being considered a poor prognostic factor in this disease. In previous reports, we have shown that this protein induces cancer stem cell and invasive properties to basal-like breast cancer cells. Here, we clarify the downstream signaling pathways that are triggered by P-cadherin to mediate these effects.

We demonstrated that P-cadherin inhibition led to a significant decreased adhesion of cancer cells to the basement membrane substrate laminin, as well as to a major reduction in the expression of the laminin receptor &alpha;6β4 integrin. Remarkably, the expression of this heterodimer was required for the invasive capacity and increased mammosphere forming efficiency induced by P-cadherin expression. Moreover, we showed that P-cadherin transcriptionally up-regulates the &alpha;6 integrin subunit expression and directly interacts with the β4 integrin subunit. We still showed that P-cadherin downstream signaling, in response to laminin, involves the activation of focal adhesion (FAK), Src and AKT kinases. The association between the expression of P-cadherin, &alpha;6β4 heterodimer and the active FAK and Src phosphorylated forms was validated *in vivo*.

Our data establish that there is a crosstalk between P-cadherin and the laminin receptor &alpha;6β4 integrin signaling pathway, which link has never been previously described. The activation of this heterodimer explains the stem cell and invasive properties induced by P-cadherin to breast cancer cells, pointing to a new molecular mechanism that may be targeted to counteract the effects induced by this adhesion molecule.

## INTRODUCTION

Cadherin molecules have a major role in tumor progression. A significant example is E-cadherin, for which a tumor suppressor function was already described in the majority of human cancer models. In fact, one of the first steps in the metastatic cascade is the loss or downregulation of E-cadherin expression or function by cancer cells, and it is known that mutations of its codifying gene (*CDH1*) increases the risk to develop particular types of breast and gastric cancers [1]. P-cadherin, on the other hand, has a promoting effect in several solid tumors, including the ones from pancreas, prostate, colon and breast [1-6]. Indeed, we have previously demonstrated that P-cadherin is a poor prognostic factor for breast cancer patients, being significantly associated with lack of cell differentiation and high grade carcinomas [5, 7]. Its expression was found to be up-regulated in the particularly aggressive basal-like molecular subtype [5, 8, 9], and *in vitro* studies from our group have shown that P-cadherin increases cell invasion and motility [10], as well as induces the secretion and activation of metalloproteinases to the extracellular matrix (ECM) [11]. Recently, we further described its capacity to induce stem cell properties in basal-like breast cancer models [12].

The maintenance of stem cell activity requires signaling mediated by the ECM and by ECM receptors, also known as integrins [13]. Integrins play a major role in the integration of signals form the external microenvironment and from the cell internal milieu. In the normal breast, the basal/myoepithelial cells are in direct contact with the basement membrane, which is composed of a complex mixture of ECM molecules that contribute to the survival and adhesion signaling of epithelial cells and to the maintenance of the stem cell niche within this tissue. Interestingly, P-cadherin is also expressed by this basal cell layer and we have previously demonstrated that it is co-expressed with α6 integrin ECM-adhesion receptor (or CD49f) in a population of cells that show stem-like properties [12].

Alterations in the ECM or in the integrin expression are implicated in the initiation and progression of breast cancer [13, 14]. For example, ECM remodeling and integrin activation assist in the malignant transformation of cells in the primary site, as well as in the activation of quiescent cells in distant metastatic sites, such as the bone, liver, lung and brain [15-18]. In normal breast, the basement membrane has a crucial role in limiting tumor progression, being composed mainly by collagen type-IV and several laminins [19]; but, in cancer, elevated expression of laminin is considered a poor prognostic factor [19, 20]. In fact, abnormal overexpression of laminin-332 (formerly known as laminin 5) is present in the migrating edge of the tumor mass and the expression of laminin receptors are believed to promote invasion of breast cancer cells [19, 21]. Although several integrins recognize laminin substrates, the α6 integrins (α6β1 and α6β4) are the major receptors that contribute to breast cancer progression [22, 23]. Thus, the role of the heterodimer α6β4 in tumor progression has been extensively investigated. Aberrant activation of the α6β4 receptor is implicated in cell survival, migration and invasive potential [22-25]. Interestingly, the expression of the β4 integrin subunit is associated with poor breast cancer patient prognosis [20, 26] and specifically with the basal-like molecular subtype [26]. Although mice in which β4 integrin was inactivated in the mammary gland have a normal breast development [27], this integrin subunit was found to be crucial for breast cancer progression [28]. Furthermore, overexpression of the α6 integrin subunit was found in invasive breast carcinomas correlating with decreased overall patient survival [29], being an important breast stem cell marker in both mice and humans [30-33]. A major role has been also proposed for β1 integrin subunit in the normal development of the murine breast, being an important marker of normal murine stem cells [34] and regulating the ability of the stem cells to self-renew and properly differentiate during pregnancy [35]. This integrin molecule has also an important role in tumorigenesis, since the disruption of this integrin in the mammary gland of a polyomavirus middle T antigen (PyMT) transgenic mouse model completely blocked tumor formation [36].

Thus, the crosstalk between cell-cell and cell-ECM adhesion complexes reflects a highly integrated intracellular network. Integrin and cadherin adhesion molecules often cooperate, activating the same signaling pathways and eliciting similar cellular functions that are part of a larger adhesive structure. In cancer, an association of cadherins and integrins can originate complexes that mediate important oncogenic responses, often through interaction with other transmembrane proteins, such as growth factor receptors. Several reports focus on the association of E-cadherin with integrin molecules [37-40], but no interaction between P-cadherin and integrin molecules has ever been described. P-cadherin is well described as having a role in cell-cell interactions; however, its role in cell-ECM interaction remains completely unknown.

The aim of this study was to find out if the P-cadherin-induced aggressive features were dependent on microenvironment signals, in particular, the ECM components and integrin receptors. Herewith, we demonstrated that P-cadherin is needed for breast cancer cell adhesion to specific ECM components. The expression of the laminin receptor α6β4 integrin was found to depend on P-cadherin expression. Moreover, this integrin heterodimer was involved in the mammosphere formation ability induced by P-cadherin expression in breast cancer cells. A new signaling mechanism triggered by P-cadherin is described that involves the activation of FAK, Src and AKT kinases in response to laminin.

## RESULTS

### P-cadherin expression potentiates the adhesion of basal-like breast cancer cells to laminin

The role of P-cadherin as a cell-cell adhesion molecule is well documented; however, its role in cell-ECM adhesion is completely unknown. Thus, the adhesion of cancer cells to several ECM components typically implicated in tumour progression was assessed.

**Figure 1:**
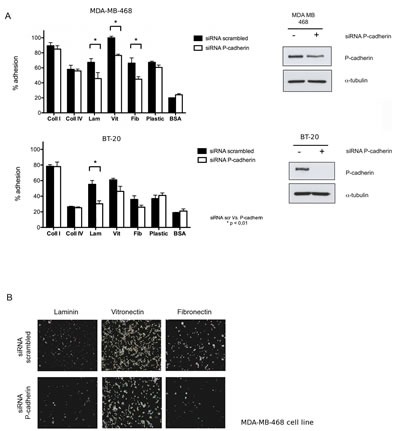
Adhesion of basal-like breast cancer cell lines to extracellular matrix (ECM) components is dependent on P-cadherin expression (A) Inhibition of P-cadherin expression in MDA-MB-468 cells decreased % adhesion to laminin-332, vitronectin and fibronectin (adhesion time = 20 min). A significant decrease in cell adhesion was also observed for BT-20 cell line in laminin (adhesion time = 30 min); (B) Bright field images of MDA-MB-468 cells in the tissue culture plate coated with ECM substrates after the adhesion assay. Cells were fixed and the nuclei stained with crystal-violet. (Coll I – collagen I, Coll IV – collagen IV, Lam – laminin 332, Vit – vitronectin, Fib – fibronectin, BSA – bovine serum albumin, negative control).

The basal-like epithelial breast cancer cell lines MDA-MD-468 and BT-20 were used as model systems, which are characterized by the positive expression of E-cadherin, negativity for hormone-receptors, lack of HER-2 amplification and high levels of basal markers, including high expression of EGFR and P-cadherin. Transient knock-down of P-cadherin was performed by siRNA (60% inhibition in MDA-MB-468 and 82% inhibition in BT-20, at the protein level) and adhesion to collagen type-I, collagen type-IV, laminin-332, vitronectin and fibronectin was measured by the crystal-violet adhesion assay (Figure 1).

MDA-MB-468 and BT-20 control cells preferentially adhered to collagen type-I and vitronectin, followed by a moderate adhesion to collagen type-IV, laminin-332 and fibronectin. Adhesion of both cell lines to plastic was approximately 70% for MDA-MB-468 cells and 40% for BT-20 cells (Figure 1A). When P-cadherin was inhibited in MDA-MB-468 cells, adhesion to laminin-332, vitronectin and fibronectin was significantly reduced by about 20%, whereas adhesion to the collagen molecules (type I and IV) was not affected (Figure 1A and 1B). For the BT-20 cell line, a significant 25% decrease in the adhesion to the laminin substrate was specifically observed after P-cadherin knock-down (Figure 1A).

### P-cadherin regulates the expression of the laminin receptor α6β4 integrin in breast cancer cells

Since P-cadherin regulates the adhesion of cancer cells to specific ECM components, we set out to investigate whether this effect was mediated by any alteration in the expression of integrins, the main receptors involved in ECM-cell adhesion. β1 integrin is a major component of most integrin heterodimers, recognizing several ECM components, including laminin, vitronectin and fibronectin. Vitronectin and fibronectin are also recognized by RGD integrin receptors, specifically containing β3, αV and α5 integrin subunits [41]. Laminin is mainly recognized by integrins that contain α6 and β4 subunits, which bind exclusively to this ECM substrate, and it is described as having important tumor promoting effects in breast cancer [22-25]. Based on this knowledge, we analyzed the surface expression of β1, β3, β4, α5, α6, and αV integrin subunits by flow cytometry in the MDA-MB-468 and BT-20 basal-like breast cancer models, with or without the silencing of P-cadherin transcripts by siRNA.

**Figure 2:**
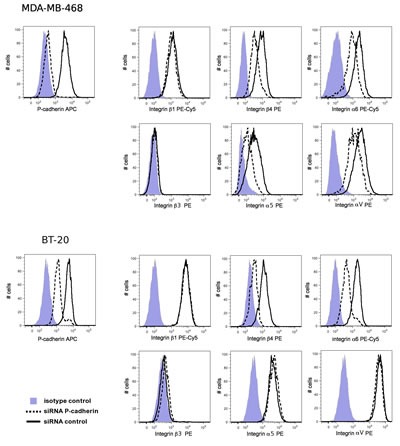
P-cadherin knock-down reduces integrin α6 and β4 expression in MDA-MB-468 and in BT-20 cells Cell surface expression of P-cadherin and integrin molecules was analyzed by flow cytometry. The median intensity of integrins α6 and β4 stain was decreased upon P-cadherin knock-down. No effect was observed in the expression of the integrin subunits β1 or β3.

As shown in Figure 2, the inhibition of P-cadherin expression had no effect in the expression of β1 or β3 integrins in both cell lines analyzed. Despite having found a reduction in the α5 and αV integrin subunits in MDA-MB-468 cells, no differences were found in the cell surface expression of these integrins in BT-20. Noteworthy, P-cadherin knock-down caused a reduction in the cell surface expression of the α6 and β4 integrin subunits in both cell lines (Figure 2A). Interestingly, α6 and β4 form a heterodimer (also known as hemidesmosome in normal cells) that recognizes the major component of the basement membrane, laminin-332, for which we demonstrated that adhesion was impaired upon P-cadherin knock-down in both cell lines analyzed (Figure 1A).

**Figure 3:**
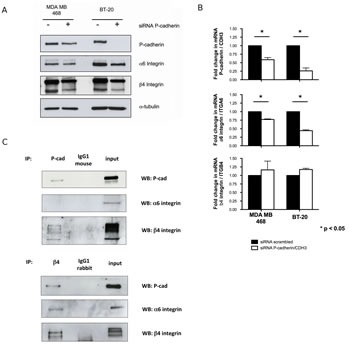
P-cadherin controls the expression of α6β4 integrin heterodimer in basal-like breast cancer cell lines Transient inhibition of the P-cadherin/CDH3 gene in MDA-MB-468 and BT-20 cells leads to a decrease in the expression of α6 and β4 integrin subunits, measured by western blot (A); the mRNA level of α6 integrin/ITGA6 is decreased upon P-cadherin silencing, whereas β4 integrin/ITGB4 mRNA level is unaffected (B); Co-immunoprecipitation experiments show that P-cadherin directly interacts with the β4 integrin subunit, but not with the α6 integrin subunit in these cells (the BT-20 cell line is represented).

Furthermore, the expression of α6 and β4 subunits was also evaluated by immunofluorescence and immunoblotting, confirming a decrease in the total amount of these integrins in breast cancer cells after P-cadherin knock-down (Figure 3A and Supplementary Figure 1). The decrease in the α6 integrin protein expression is accompanied by a decrease in the mRNA levels of the α6 integrin/*ITGA6* gene, whereas the mRNA levels of the β4 integrin/*ITGB4* gene are not affected (Figure 3B). Since lateral integrin-cadherin associations are known to occur [37, 39, 42], we also tested the existence of a physical interaction between P-cadherin and α6β4 integrin; the β4 integrin subunit and P-cadherin were able to co-immunoprecipitate (Figure 3C).

### P-cadherin and the α6 integrin confer stem cell properties and invasive features to breast cancer cells

Since P-cadherin expression impacts cell-ECM adhesion and clearly modifies integrin α6β4 expression in breast cancer cells, we set out to study if this integrin heterodimer was also implicated in the aggressive properties that have been previously ascribed to P-cadherin, namely, the invasive capacity and the mammosphere forming ability. Furthermore, to clarify the crosstalk between P-cadherin and α6β4 integrin, the effect of both α6 and β4 integrin subunits in the expression levels of P-cadherin were also studied.

**Figure 4:**
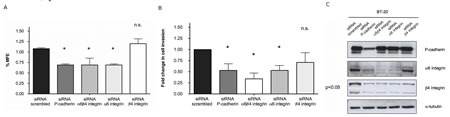
Inhibition of α6β4 integrin decreases the mammosphere forming efficiency (MFE) in breast cancer cells to the same extent as inhibition of P-cadherin (A) In the same way, the invasion capacity of these cells in matrigel was severely compromised when P-cadherin or α6β4 integrin were knocked-down (B); although the expression of the α6 and β4 subunits is decreased upon P-cadherin knock-down, the expression of P-cadherin is not affected after the inhibition of either α6 integrin or β4 integrin or both integrins at the same time (C). Results for the BT-20 cell line are shown in the figure. Similar results were observed for the other basal-like cell line, MDA-MB-468.

Inhibition of α6β4 in breast cancer cells significantly decreased the mammosphere forming efficiency (MFE), as well as the invasion capacity, precisely in the same magnitude as the one induced by P-cadherin inhibition (Figure 4A and 4B). Importantly, α6 integrin inhibition alone showed the same impact in MFE and in the invasion potential as the inhibition of P-cadherin or the repression of the α6β4 heterodimer. However, inhibition of the β4 integrin subunit in breast cancer cells did not show a statistically significant impact in these functional properties (Figure 4A and 4B). These results indicate that P-cadherin downstream signaling effects could be primarily dependent on the α6 integrin subunit function.

It is also interesting to note that, while P-cadherin knock-down caused a reduction in α6 and β4 integrin subunits, the opposite was not true (Figure 4C). The inhibition of α6 and/or β4 integrins showed no effect in P-cadherin expression. Nonetheless, α6 integrin knock-down led to a decrease in the expression of its partner, the β4 integrin subunit, pointing that P-cadherin may in fact be controlling the α6 subunit expression, which in turn controls the β4 subunit, as already shown by Klinowska and colleagues [27]. In summary, the functional properties attributed to P-cadherin expression were only affected when the α6 integrin subunit or the α6β4 integrin heterodimer were inhibited; the inhibition of β4 integrin subunit had no effect in MFE and invasion.

### P-cadherin overexpressing cells have increased adhesion to laminin as well as increased mammosphere forming ability and these properties are dependent on α6β4 integrin expression

**Figure 5:**
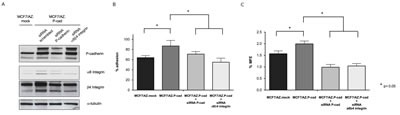
P-cadherin overexpression induces increased adhesion to laminin-332 and increased mammosphere forming capacity in a α6β4 integrin dependent manner P-cadherin overexpression in MCF7/AZ cells (MCF7/AZ.P-cad) induces the expression of α6 and β4 integrin subunits (Vs. MCF7/AZ.mock cells), measured by western-blot (A); P-cadherin overexpression also induced increased adhesion to laminin-332, evaluated by the adhesion assay (adhesion time = 30min) (B) and increased mammosphere forming efficiency (MFE) (C). Inhibition of the α6β4 integrin heterodimer in the P-cadherin overexpressing cells restored the levels of adhesion to the control levels, and strongly inhibited the MFE in these cells. P-cadherin expression was unaffected by α6β4 integrin knock down.

The previous results indicated that there could be a crosstalk between two adhesion molecules: P-cadherin and α6 integrin. Thus, to further explore the role of α6 integrin and its partner, β4 integrin, in the functional properties mediated by P-cadherin, we analyzed the cell-laminin adhesion capacity and the MFE of a breast cancer cell line constitutively overexpressing P-cadherin (MCF7/AZ.P-cad) and compared these properties with control cells, which have low levels of P-cadherin (MCF7/AZ.mock). P-cadherin expression was accompanied by an increase in the expression of both, the α6 integrin subunit, as well as the β4 integrin subunit (Figure 5A). Importantly, P-cadherin upregulation led to an increase in the adhesion of MCF7/AZ.P-cad cells on top of a laminin coated surface (Figure 5B) and increased the mammosphere forming ability of these cells (Figure 5C). These effects were mediated, at least partially, by α6β4 integrin expression, since the levels of this integrin heterodimer are increased in P-cadherin overexpressing cells (Figure 5A); when both integrin subunits were simultaneously knocked-down in MCF7/AZ.P-cad cells, these functional properties were significantly reduced (Figure 5). Once more, P-cadherin levels were not affected by α6β4 integrin knock down, indicating that these integrin molecules are most likely acting downstream of P-cadherin activation (Figure 5A).

### Integrin signaling in response to laminin-332 is dependent on P-cadherin expression

**Figure 6:**
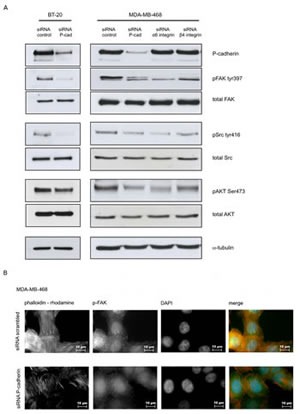
P-cadherin signaling in response to laminin involves FAK/Src activation Analysis of integrin downstream signaling molecules in breast cancer cell lines was performed after adhesion to laminin-332 (20 min for MDA-MB-468 and 30 min for BT-20) (A); The number of stress fibers (F-actin was stained with phalloidin-rhodamine) and focal adhesions/contacts (stained with pFAK Tyr397 – Alexa 488) is reduced by P-cadherin knockdown in MDAB-MB-468 cells grown on top of laminin (B). The same result was found for BT-20 cell line.

The previous data established a cross-talk between P-cadherin and α6β4 integrin in basal-like breast cancer cell models. We therefore studied whether P-cadherin could affect the main signaling molecules downstream of the α6β4 integrin receptor in cancer cells, when these were grown on top of a laminin substrate. The activation of the integrin related kinases FAK and Src was studied by immunoblotting after cell adhesion to this substrate. We found that P-cadherin inhibition in breast cancer cells reduced p-FAK Tyr397 and p-Src Tyr416 levels (Figure 6A). Notably, the p-FAK Tyr397 reduction was also detected by immunofluorescence in both cell lines studied (Figure 6B). Furthermore, activation of AKT was also affected, shown by a reduction in level of p-AKT Ser473 (Figure 6A). Altogether, these results indicate that FAK and Src activation in response to laminin is dependent on P-cadherin expression in basal-like breast cancer cells.

We also investigated if the cancer cell phenotype was affected in cells grown on top of the substrate for α6β4 integrin. Thus, we analyzed the cytoskeleton microfilaments by phalloidin staining by fluorescence microscopy in breast cancer cells adhered to laminin coated coverslips (Figure 6B). We found that control cells (scrambled transfected) had more stress fibres and appeared more flattened than cancer cells with P-cadherin knock-down. The stress fibres provide the cytoskeletal tension which is required for focal adhesion formation in laminin, indicating a strong adhesion to the ECM substrate. Staining with an antibody for p-FAK Tyr397 allowed the identification of focal adhesions and sites of cell-to-cell contacts. Both focal adhesions and cell-cell contacts were decreased in P-cadherin depleted cells (Figure 6B). In summary, P-cadherin has a role in eliciting cell shape changes associated with adhesion to the ECM.

### Assessment of P-cadherin/α6β4 integrin/FAK/Src cross-talk signaling in in vivo tumor xenografts

Since the data collected *in vitro* pointed out to an activation of FAK/Src signaling in a P-cadherin dependent manner, we decided to analyze if this signaling pathway was also present in the *in vivo* setting. In order to study tumors with different P-cadherin expression levels, we have used the basal-like and P-cadherin positive MDA-MB-468 cell line and FACS to separate the top 20% P-cadherin expressing cells from the low 20% P-cadherin expressing cells (purity of sorted populations was 85-95%). These sorted cells, as well as the unsorted population, were inoculated into the subcutaneous region, under the left abdominal mammary fat pad of immune compromised mice. The tumorigenic capacity was evaluated after 30 days and tumors were characterized by immunohistochemistry for P-cadherin, α6 integrin, β4 integrin, pFAK and pSrc.

The percentage of tumors formed with the unsorted population was 66.6% (8/12 mice). The top 20% P-cadherin group of animals presented an increased tumor formation capacity (85.7%, 6/7 mice) compared to the P-cadherin low 20% group (28.6%, 2/7 mice). All the tumors formed were histologically classified as solid with infiltrative growth and extensive necrosis (Supplementary Figure 2A).

**Figure 7:**
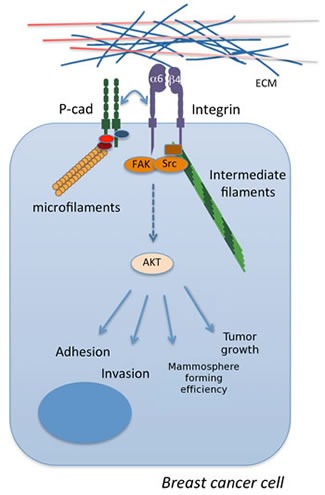
The crosstalk between P-cadherin and α6β4 integrin in basal-like breast cancer These signaling molecules cooperate leading to the phosphorylation and activation of FAK, Src and AKT, mediating tumor growth and important aggressive cancer properties *in vitro*, such as cell invasion, adhesion to laminin and mammosphere formation.

Concerning the immunohistochemical analysis, 25% (4/16) of the tumors showed high expression for P-cadherin, 31.2% (5/16) of the tumors formed were considered positive for α6 and β4 integrins, 18.7% (3/16) were positive for pFAK and 43.7% (7/16) were positive for pSrc (Supplementary Figure 2 and Supplementary Table).

Our results showed that there is a statistically significant association between the tumors with a high expression of P-cadherin (P-cad high) and the expression of α6 integrin (p=0.0027), β4 integrin (p=0.0027), pFAK (p=0.0071) and pSrc (p=0.0192) (Table 1), validating the signaling pathway previously found *in vitro*. Additionally, we were also able to find an association between α6 integrin and β4 integrin (p=0.0005, data not shown), as well as between pFAK and both integrin subunits (p=0.0179, data not shown).

**Table 1:** *In vivo* association of P-cadherin expression with the α6β4 integrin heterodimer and the activation of FAK/Src pathway Tumours were formed *in vivo* from the MDA-MB-468 cell line with different levels of P-cadherin expression and they were characterized by IHC for the expression of α6 integrin, β4 integrin, pFAK and pSrc. A statistically significant association was found between P-cadherin and the expression of α6 integrin, β4 integrin, pFAK and pSrc (Fisher's exact test)

		P-cadherin		
		High (n=4)	Low (n=12)	p value
α6 integrin	Positive (n=5)	4 (100%)	1 (8.33%)	0.0027
	Negative/low (n=11)	0 (0%)	11 (91.67%)	
β4 integrin	positive (n=5)	4 (100%)	1 (n=8.33%)	0.0027
	Negative/low (n=11)	0 (0%)	11 (n=91.67%)	
pFAK	Positive (n=3)	3 (75%)	0 (0%)	0.0071
	Negative/low (n=13)	1 (25%)	12 (n=100%)	
pSrc	Positive (n=)	4 (100%)	3 (25%)	0.0192
	Negative/low (n=)	0 (0%)	9 (75%)	

### Discussion

Cadherins are classically seen as molecules that make a major contribution for cell-to-cell adhesion. Specifically in breast, P-cadherin expression is found in the myoepithelial cell layer, strongly contributing to the self-organization of these cells [43]. Notably, this basal layer of the mammary epithelium is also enriched in molecules involved in the adhesion of epithelial cells to the ECM, namely in integrin molecules, such as α6β1 and α6β4. In normal cells, the later heterodimer is known as hemidesmosome and it is the receptor for laminin, the major component of the basement membrane.

In breast cancer, P-cadherin molecule appears upregulated in 30-40% of all diagnosed cases, being significantly associated with poor patient prognosis [5, 44]. It is known, however, that breast cancer progression involves modifications of the normal ECM, as well as oncogenic activation of integrin signaling in both primary tumors, as well as in the metastatic sites [16, 45]. Here, we established that P-cadherin is involved in the attachment of cells to ECM substrates, since its silencing rendered cancer cells significantly less able to adhere to vitronectin, fibronectin and laminin. When integrins expression was investigated, we found that P-cadherin was necessary for the appropriate expression of the integrin subunits α6 and β4. Importantly, the recognition of laminin by cancer cells has significant tumor promoting effects. For example, laminin-332 induces motility in the MCF-7 breast cancer cell line [46]. Furthermore, IHC analysis of laminin-332 in human *in situ* breast carcinomas showed that this ECM substrate is located in the myoepithelium adjacent to preinvasive cells [21, 46], that could potentially contribute to the early steps of stromal invasion. The interface zone between the tumour cells and the stroma is enriched in laminin, as well as in α6β4 integrin [21].

We have previously shown that P-cadherin induces invasion and migration of breast cancer cells [11] and plays an important role in breast tumourigenesis *in vivo* [12, 47]. The signaling pathways that contribute to this aggressive behavior are poorly understood, involving to some extent the activation of metalloproteinases and the consequent release of a soluble pro-invasive P-cadherin fragment and/or the activation of small GTPases [6, 11]. Recently, we have shown that P-cadherin has been implicated in the maintenance of stem and progenitor properties in basal-like breast cancer cells, including the self-renewal capacity and the tumorigenic ability in nude mice [12]. We also found that P-cadherin is co-expressed with α6 integrin in breast cancer cells [12], a marker of the stem/progenitor phenotype present in the mouse and human breast [30-33]. In the present work, we explored further this association, showing that there is a crosstalk between both adhesion molecules. P-cadherin is acting upstream of a major signaling pathway that involves the activation of α6 integrin and its partner, the β4 subunit. As a consequence of the adhesion of cancer cells to laminin surface, the activation of the α6β4 heterodimer would lead to Src and FAK activation in a P-cadherin dependent manner. In fact, in the present study, we showed that P-cadherin knock-down reduces FAK and Src phosphorylation *in vitro* and an association was found between P-cadherin with the α6β4 heterodimer and FAK/Src activation *in vivo*. Importantly, it has been reported that α6β4 integrin promotes survival and invasion by activating the PI3K/Akt pathway [24, 48] and notably, in our work, a reduction was also found in AKT activation in P-cadherin silenced cells.

It was previously found that α6 integrin activation induces P-cadherin transcription [49], further supporting the idea that P-cadherin could cooperate with α6 integrin signaling. However, our work revealed that P-cadherin and α6 integrin do not directly interact. Rather, P-cadherin expression seems to control the transcription of α6 integrin subunit, since silencing of P-cadherin leads to a decrease in the α6 integrin mRNA levels. On the other hand, β4 integrin mRNA levels were unaffected by P-cadherin inhibition, but the physical interaction between P-cadherin and β4 integrin subunit points to a possible regulation at a post-transcriptional level. In fact, the absence of β4 integrin after P-cadherin silencing may be due to the downregulation of its unique partner, the α6 integrin, hence blocking the formation of the heterodimer, as already shown by Klinowska and colleagues [27].

β1 integrin is also a partner of α6 integrin subunit recognizing laminin, being also essential for the correct development of the mammary epithelium, and regulating the ability of the mammary stem cells to self-renew and differentiate properly [35]. Despite not having found any alteration in the β1 integrin levels upon P-cadherin inhibition, we do not exclude the possibility that β1 integrin subunit is also implicated in the maintenance/acquisition of cancer stem cell and invasive properties, as this is the other major partner of α6 integrin, constituting an important laminin receptor.

Additionally, although the cell morphology was not severely affected by P-cadherin knock-down and cells clearly maintained an epithelial phenotype, we found that the number of cell-to-cell contacts and the number of focal adhesions to laminin was clearly reduced upon P-cadherin inhibition. It is possible that α6β4 integrin, and the subsequent FAK/Src kinase activity, may also be contributing to the stem/progenitor characteristics. It was shown that FAK deletion in the murine mammary gland suppressed tumorigenesis by decreasing the number of cancer stem cells (CD24^+^CD29^+^CD61^+^ and ALDEFLUOR^+^ populations) [50]. Notably, FAK activation allows for the survival of cells in anchorage-independent conditions [51], which may explain why integrin knock-down, as well as P-cadherin knock-down, reduced survival of cells growing as suspension colonies in which the ECM is present within the mammosphere.

Thus, the poor patient prognosis found in P-cadherin overexpressing breast cancer cases [10] may be related, at least partially, to the fact that this cadherin enables cells to respond to integrin signaling, promoting an oncogenic response. Importantly, strategies to inhibit P-cadherin could lead to a decrease in integrin activation and potentially oppose the oncogenic signaling mediated by laminin and its receptor. Since P-cadherin up-regulation is also found in *in situ* stages of breast cancer development [7], it is possible that it may already be contributing to the changes in integrin signaling in the early stages of breast cancer development.

In conclusion, our results show that P-cadherin controls the cell-to-laminin adhesion, by modulating the expression and the activation of the α6β4 integrin heterodimer. Moreover, P-cadherin and α6β4 integrin have oncogenic signaling pathways that cooperate and cross-talk, in order to induce cancer cell invasion and survival in anchorage independent conditions. These results are particularly relevant, since they provide a new link for P-cadherin and the tumor microenvironment and a new molecular mechanism explaining P-cadherin aggressive behavior in breast carcinomas.

## METHODS

### Ethics statement

Investigation has been conducted in accordance with the ethical standards and according to the Declaration of Helsinki and according to national and international guidelines and has been approved by the authors’ institutional review board.

### Cell culture

Human breast cancer cell lines MDA-MB-468 and BT-20 were obtained from ATCC (American Type Culture Collection, Manassas, VA). These cell lines were grown in DMEM supplemented with 10% fetal bovine serum (FBS) and 1% antibiotic solution (penicillin–streptomycin) (Invitrogen, Carlsbad, CA). The human breast cancer cell line MCF-7/AZ was obtained from a collection developed in the laboratory of Prof. Marc Mareel (Ghent University Hospital, Belgium), which was genetically manipulated to stably overexpress P-cadherin (MCF-7/AZ.Pcad). The control cell line (MCF-7/AZ.mock) shows low P-cadherin levels, identical to the parental cell line [10]. These cell lines were cultured in DMEM/F12 supplemented with 10% FBS and 1% antibiotic solution (penicillin–streptomycin) (Invitrogen). All cells were routinely cultured in a humidified atmosphere with 5% CO_2_ and at 37°C and were used in experiments when reached 70–80% confluence.

### RNA knock-down of P-cadherin and integrin molecules

Gene silencing was conducted by siRNA sequences targeting specific genes. P-cadherin (*CDH3* gene) target sequence: AAGCCTCTTACCTGCCGTAAA, Integrin ☐6 (*ITGA6* gene) target sequence: CAGGGTAATAAACTTAGGTAA, Integrin ☐4 (*ITGB4* gene) target sequence: GTGGATGAGTTCCGGAATAAA. All siRNA sequences were obtained from Qiagen (Hilden, Germany). Cell transfection was carried out using HiPerFect transfection reagent (Qiagen) in a final concentration of 5 nM siRNA, according to the manufacturer's instructions. Optimal silencing of the target genes was achieved at 48hafter transfection, which was confirmed by immunoblot analysis. A siRNA scrambled sequence was included as a control (Qiagen).

### Adhesion assay to ECM substrates

Cell adhesion assay was performed by the crystal violet assay in 96-well microtiter plates coated with laminin-332 (Sigma, St. Louis, MO), fibronectin (Sigma), vitronectin (BD Biosciences, San Diego, CA), type-I or type-IV collagen (Sigma) (5µg/ml) overnight at 4°C. Subsequently, plates were washed three times in PBS and non-specific-binding sites were blocked by adding 0.5% BSA (w/v) in PBS containing Pen/Strep (Invitrogen) for 2h at 37°C. Once washed again with PBS, 100µl of cells (10^6^ cells/ml) were seeded in serum-free medium for 20 minutes (for MDA-MB-468 cell line) or 30 minutes (for BT-20 cell line). Thereafter, the plates were washed with PBS to remove non-adherent cells, and the attached cells were fixed with acetone:methanol (1:1) for 10 minutes at 4°C. Cell adhesion was determined following the colorimetric method described by Busk [52]. The absorbance was measured at 570nm with a microplate reader. The attachment of cells to wells coated with 1mg/ml of poly-L-Lys (Sigma) and fixed with 4% paraformaldehyde before aspiration was defined as 100% of adhesion.

### Flow Cytometry analysis

Cells were harvested with versene/0.48mM EDTA (Invitrogen). Detached cells were washed with PBS supplemented with 0.5% FBS and re-suspended in the stain buffer (2mM EDTA + 0.5% bovine albumin in PBS). A single cell suspension was labeled by fluorescence-conjugated antibodies at a concentration of 1 to 10 in stain buffer: PE-Cy5-conjugated Integrin β1 (CD29), PE-Cy5-conjugated Integrin α6 (CD49f) or PE-conjugated Integrin β4 (CD104). These antibodies were obtained from BD Biosciences (San Diego, CA). P-cadherin monoclonal antibody APC-conjugated was obtained from R&D (Minneapolis, MN) and used at the same concentration as above. A live-dead stain (Invitrogen) and the primary antibodies or the respective isotype controls (BD Biosciences) were incubated at 4°C, in the dark, for 15 minutes. The labeled cells were then washed in the stain buffer and analyzed on a LSR-II or FACS Canto-II (BD Biosciences).

### Immunofluorescence microscopy

Different cell lines were seeded on top of glass coverslips coated with laminin-332 (Sigma). Cells were fixed with 4% paraformaldehyde, permeabilized with 0.1% Triton X-100 and blocked with 5% BSA before staining. The following primary antibodies were used for immunofluorescence: FITC-conjugated α6 integrin (CD49f) (1:10, BD Biosciences), PE-conjugated β4 integrin (1:10, CD104) (BD Biosciences) and p-FAK tyr397 (1:200 dilution, Cell Signaling). To visualize p-FAK, anti-rabbit Alexa-488 (1:1000, Invitrogen) was incubated on slides for 30 minutes. F-actin was detected by staining with phalloidin conjugated to rhodamine (Invitrogen) at a dilution of 1:1000. Cells were visualised using a Zeiss Imager Z.1 microscope (Zeiss, Welwyn Garden City, UK) and representative photos were acquired using the associated software: Photoshop and Illustrator (both CS4; Adobe).

### Immunoblotting analysis

After performing the adhesion assay over a laminin coated surface (6 wells plate, BD Biosciences), cells were lysed with PBS containing 1% Nonidet-P40 (NP40, Sigma-Aldrich, St. Louis, MO) and phosphatase (Sigma) and protease inhibitors (Roche Diagnostics Gmbh, Mannheim, Germany). Protein concentration was determined by Bio-Rad protein assay (Bio-Rad, Richmond, CA) and 30 µg of total protein was resolved on a 10% denaturing polyacrylamide gel and transferred onto a nitrocellulose membrane (Amersham Pharmacia Biotech, Piscataway NJ). After blocking nonspecific binding with 5% non-fat dry milk (for non-phosphorylated protein detection) or 5% BSA (for phosphorylated protein detection) in PBS containing 0.5% Tween 20, each membrane was incubated for 1 hour at room temperature with each of the following primary antibodies: anti-P-cadherin (1:500, clone 56, BD Transduction), anti-α6 integrin (1:1000, Sigma-Aldrich), anti-β4 integrin (1:2000, Santa Cruz Biotechnology), anti-pSrc Tyr 416 (1:1000, Cell Signalling, Danver, MA), anti-total Src (1:1000, Cell Signalling), anti-pFAK Tyr 397 (1:1000, Cell Signalling), anti-total FAK (1:500, BD Transduction), anti-pAKT Ser 473 (1:2000, Cell Signalling) and anti-AKT1/2 (1:500, Santa Cruz Biotechnology). Anti-α-tubulin (1:10000, clone DM1A, Sigma-Aldrich) was used in all the blots as a loading control. Secondary antibodies were peroxidase-conjugated from Santa Cruz Biotechnology. Immunoreactive proteins were detected by enhanced chemiluminescence detection kit (Amersham, GE Healthcare, Uppsala, Sweeden) and exposure to Hiperfilm ECL (Amersham).

### Mammosphere assay

Monolayer cells were enzymatically detached with 0.125% trypsin-EDTA (Sigma-Aldrich), manually disaggregated with a 25-gauge needle to a single-cell suspension and suspended in cold PBS. Cells were plated at 500/cm^2^ in non-adherent culture conditions, in flasks coated with 1.2% poly-2-hydroxyethylmethacrylate / 95% ethanol (Sigma). Cells were grown, for 5 days, in DMEM/F12 containing B27 supplement, 500 ng/ml hydrocortisone, 40 ng/ml insulin, 20 ng/ml EGF and maintained in a humidified incubator at 37°C and 5% (v/v) CO_2_. Mammosphere forming efficiency (MFE) was calculated as the number of mammospheres (≥50 μm) formed divided by the total number of cells initially plated, being expressed as a percentage.

### Invasion assay

Matrigel invasion assay was performed according to manufacturer's instructions (BD Biosciences). Briefly, transwell chambers with polycarbonate membrane filters (6.5 mm diameter, 8 μm pore size) were coated with 20 μL of a Matrigel solution. 3.5x10^4^ BT-20 cells or MCF7/AZ cells were added to the upper compartment of the chamber. The lower compartment was filled with DMEM medium supplemented with 10% FBS and 1% antibiotic solution (penicillin–streptomycin) (Invitrogen). After 24 or 48 hours of incubation (BT20 or MCF7/AZ, respectively) at 37°C, 5% CO_2_, the upper surface of the filter was washed with serum-free DMEM and cleared from non-migratory cells with a cotton swab. The remaining (invasive) cells at the lower surface of the filter were fixed with cold methanol and stained with 4′, 6-diamidino-2-phenylindole (DAPI, Sigma-Aldrich, 0.4 mg/mL). Invasive cells were scored by counting the whole filter with a fluorescence microscope, at 200x magnification.

### Real-time RT-PCR

After transfection with siRNAs, the RNA was extracted using Qiagen RNeasy kit (Qiagen, USA). Concentration was determined in a ND-1000 spectrometer (Nanodrop) and 1 μg of total RNA was converted to cDNA using a reverse-transcriptase RT enzyme (Invitrogen, USA). P-cadherin/*CDH3*, α6 Integrin/*ITGA6* and β4 Integrin/*ITGB4* TaqMan probes (Applied Biosystems, USA) were used to specifically recognize the corresponding cDNA sequences, which were amplified for 40 cycles (Applied Biosystems 7500). A TaqMan probe for *GAPDH* was also used as a housekeeping gene and relative gene expression was determined by normalization.

### Co-Immunoprecipitation

BT-20 cells grown in monolayer were lysed with PBS containing 1% NP40 (Sigma-Aldrich) and 2 mM calcium chloride (Sigma-Aldrich), with phosphatase (Sigma-Aldrich) and protease inhibitors (Roche Diagnostics Gmbh). 500μg of cell lysate was precleared with Protein G magnetic beads (Millipore, Temecula, CA) for 10 minutes at room temperature and then incubated overnight at 4°C with 2 μg of mouse monoclonal anti-P-cadherin (Abcam, Cambridge, UK) or rabbit polyclonal anti-β4 integrin (Santa Cruz Biotechnology) or its corresponding control isotype (IgG1 mouse or IgG1 rabbit, company, respectively). The samples were then incubated with the Protein G magnetic beads (Millipore) for 10 minutes at room temperature. The beads were washed three times with washing buffer (lysis buffer diluted 1:5, containing phosphatase and protease inhibitors, as stated above) and boiled for 5 minutes in Laemmli buffer with β-mercaptoethanol (BioRad, Hercules, CA). Samples were subjected to SDS-PAGE and immunoblotting as previously described.

### In vivo assay

The P-cadherin positive cell line MDA-MB-468 was used to induce tumors in immunocompromised mice. This cell line was sorted in a BD FACS Aria II, according to P-cadherin expression (R&D antibody), into two subpopulations: top 20% P-cadherin and low 20% P-cadherin fractions. The unsorted and the sorted cells were xeno-transplanted at 5x10^4^ cells (in 100 μl DMEM cell suspension) into the subcutaneous region, under the left abdominal mammary fat pad of 4-5 weeks old female N:NIH(s)II:nu/nu nude mice, using a 25-gauge needle. Mice were sacrificed after three months. Mice were maintained and housed at IPATIMUP Animal House, sited at the Medical Faculty of the University of Porto, in a pathogen-free environment, under controlled conditions of light and humidity.

### Immunohistochemistry

A total of 16 tumors from 26 xenografted mice were isolated and fixed in 4% formaldehyde. Immunohistochemistry (IHC) was performed with antibodies for P-cadherin (BD Biosciences) (1:50, 1 hour, RT), α6 integrin (Sigma-Aldrich) (1:50, 1 hour, RT), β4 integrin (Santa Cruz) (1:300, 1 hour, RT), pFAK Tyr 397 (Cell Signalling) (1:50, 1 hour, RT), and pSrc Tyr 416 (Cell Signalling) (1:50, 4°C, overnight).

High temperature (98°C) antigen retrieval with Tris-EDTA (P-cadherin, α6 integrin, pFAK) or citrate buffer (β4 integrin, pSrc) was performed before primary antibody incubation. The primary antibodies were detected using a secondary antibody with horseradish peroxidase polymer (Cytomation Envision System HRP; DAKO, Carpinteria, CA), using diaminobenzidine (DAB) as chromogen, according to the manufacturer's instructions.

All the markers were mainly detected at the membrane of tumor cells. Concerning P-cadherin expression, all the tumors showed more than 50% of positive cells. Thus, the tumors were evaluated according with the stain intensity, being classified into P-cad high (strong stain) and P-cad low (weak and moderate stain).

According with previous published reports, the scoring for the remaining markers was considered as follows: α6 integrin stain was classified according to the stain intensity into strong (positive) and moderate/weak (negative/low) [53]; β4 integrin was classified according to the stain extension into >= 50% (positive) and <50% (negative/low) [26]; pFAK was classified according to the stain extension into >= 5% (positive) and <5% (negative/low) [54]; pSrc was considered positive when more than 50 % of tumor cells stained positive for this marker [55].

### Statistical analysis

Adhesion, MFE, invasion and changes in mRNA expression levels were compared using two-tailed unpaired t-test. Immunohistochemical associations between the molecular markers were assessed by Pearson's correlation and Fisher's exact test. Statistical analyses were carried out using Prism GraphPad (La Jolla, CA) and a significant level of 5% was considered. Flow Cytometry data was analyzed with the FlowJo software package (Treestar, Ashland, OR, USA).

### Competing interests

The authors indicate no competing interests.

## Supplementary Figures and Tables




