# Supplementary material for: P-cadherin signals through the laminin receptor α6β4 integrin to induce stem cell and invasive properties in basal-like breast cancer cells
Source: Oncotarget. 2014 Jan 6;5(3):679–92. doi: 10.18632/oncotarget.1459 (PMC3996674; doi:10.18632/oncotarget.1459)
Supplement: Supplementary file 1 [file oncotarget-05-0679-s001.pdf]

P-cadherin signals through the laminin receptor α6β4 integrin to induce stem cell and invasive properties to basal-like breast cancer cells- Vieira et al

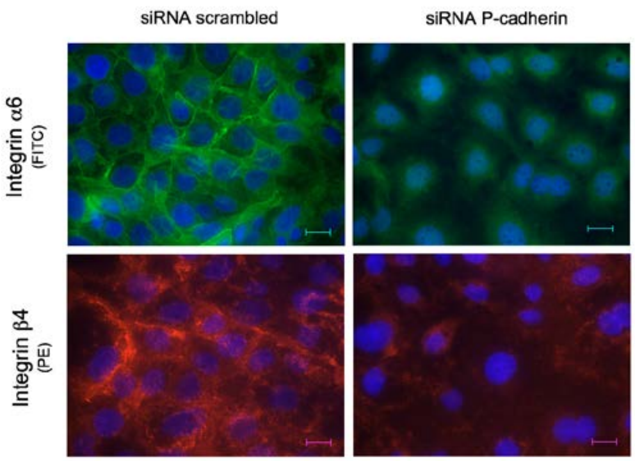

Supplementary Figure 1. Immunofluorescence staining of BT-20 cells after P-cadherin silencing revealed a decrease in α6 integrin and β4 integrin expression (scale bar=20µm). Similar results were obtained for MDA-MB-468 cell line.

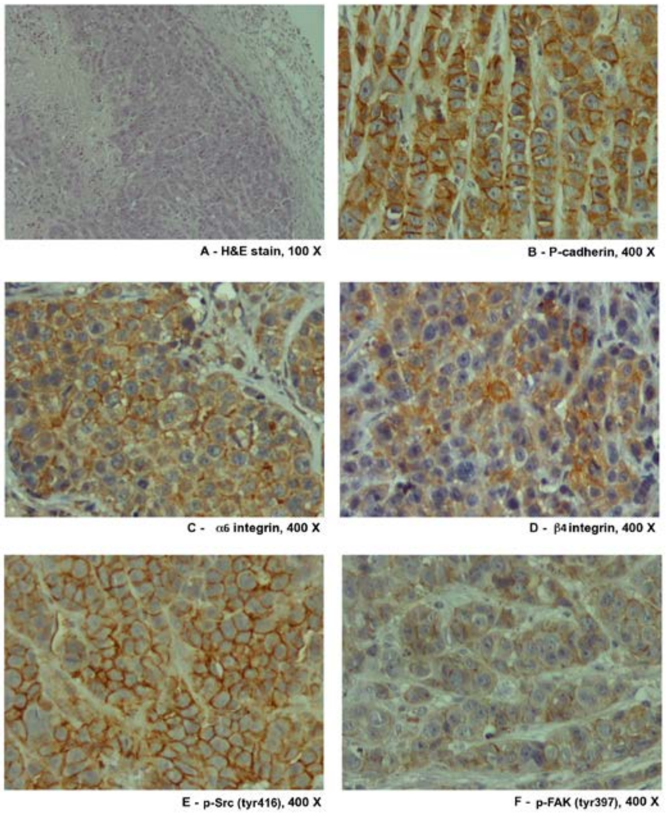

Supplementary Figure 2. Representative images of the Hematoxylin and Eosin staining (H&E) of the tumors formed *in vivo* by the MDA-MB-468 cell line in nude mice (A) and the IHC staining of a tumor case positive for P-cadherin (B), α6 integrin (C), β4 integrin (D), pFAK (E) and pSrc (F).
