# Supplementary material for: P-cadherin signals through the laminin receptor α6β4 integrin to induce stem cell and invasive properties in basal-like breast cancer cells
Source: Oncotarget. 2014 Jan 6;5(3):679–92. doi: 10.18632/oncotarget.1459 (PMC3996674; doi:10.18632/oncotarget.1459)
Supplement: Supplementary file 2 [file oncotarget-05-0679-s002.doc]

|  |  | | Tumorigenic capacity (%) | | IHC Expression | | | | |
| --- | --- | --- | --- | --- | --- | --- | --- | --- | --- |
|  |  |  |  |  | P-cadherin | α6-integrin | β4-integrin | pFAK | pSrc |
| MDA-MB-468 | Unsorted | 1 | Tumor | 8/12 (66.6%) | High | Positive | Positive | Positive | Positive |
|  |  | 2 | Tumor |  | Low | Negative/low | Negative/low | Negative/low | Negative/low |
|  |  | 3 | Tumor |  | Low | Positive | Positive/low | Negative/low | Negative/low |
|  |  | 4 | Tumor |  | Low | Negative/low | Negative/low | Negative/low | Negative/low |
|  |  | 5 | Tumor |  | Low | Negative/low | Negative/low | Negative/low | Negative/low |
|  |  | 6 | Tumor |  | High | Positive | Positive | Positive | Positive |
|  |  | 7 | Tumor |  | Low | Negative/low | Negative/low | Negative/low | Negative/low |
|  |  | 8 | Tumor |  | Low | Negative/low | Negative/low | Negative/low | Negative/low |
|  |  | 9 | No tumor |  | - | - | - | - | - |
|  |  | 10 | No tumor |  | - | - | - | - | - |
|  |  | 11 | No tumor |  | - | - | - | - | - |
|  |  | 12 | No tumor |  | - | - | - | - | - |
|  | P-cad top 20% | 1 | Tumor | 6/7 (85.7%) | Low | Negative/low | Negative/low | Negative/low | Positive |
|  |  | 2 | Tumor |  | Low | Negative/low | Negative/low | Negative/low | Positive |
|  |  | 3 | Tumor |  | Low | Negative/low | Negative/low | Negative/low | Negative/low |
|  |  | 4 | Tumor |  | High | Positive | Positive | Positive | Positive |
|  |  | 5 | Tumor |  | Low | Negative/low | Negative/low | Negative/low | Negative/low |
|  |  | 6 | Tumor |  | Low | Negative/low | Negative/low | Negative/low | Positive |
|  |  | 7 | No Tumor |  | - | - | - | - | - |
|  | P-cad low 20% | 1 | Tumor | 2/7 (28.6%) | High | Positive | Positive | Negative/low | Positive |
|  |  | 2 | Tumor |  | Low | Negative/low | Negative/low | Negative/low | Negative/low |
|  |  | 3 | No tumor |  | - | - | - | - | - |
|  |  | 4 | No tumor |  | - | - | - | - | - |
|  |  | 5 | No tumor |  | - | - | - | - | - |
|  |  | 6 | No tumor |  | - | - | - | - | - |
|  |  | 7 | No tumor |  | - | - | - | - | - |

**Supplementary Table – *In vivo* xenograft experiment using the MDA-MB-468 cell line. The unsorted population, the top 20% P-cadherin high population and the low 20% P-cadherin population were evaluated for the tumorigenic capacity in nude mice (50.000 cells/mouse). Evaluation of the expression of the markers P-cadherin, α6 integrin, β4 integrin, pFAK and pSrc was performed in the tumours formed.**
